# The mitochondrial DNA T16189C polymorphism and HIV-associated cardiomyopathy: a genotype-phenotype association study

**DOI:** 10.1186/1471-2350-10-37

**Published:** 2009-04-27

**Authors:** Gasnat Shaboodien, Mark E Engel, Faisal F Syed, Joanna Poulton, Motasim Badri, Bongani M Mayosi

**Affiliations:** 1 The Cardiovascular Genetics Laboratory, Hatter Institute for Cardiovascular Research, Department of Medicine, Groote Schuur Hospital, Cape Town, South Africa; 2 University of Cape Town, Cape Town, South Africa; 3 Department of Cardiology, University of Newcastle-upon-Tyne, Newcastle, UK; 4 The James Cook University Hospital, Middlesbrough, TS4 3BW, UK; 5 Nuffield Department of Obstetrics and Gynaecology, University of Oxford, Oxford, UK; 6 The Women's Centre, John Radcliffe Hospital, Oxford OX3 9DU, UK

## Abstract

**Background:**

The mitochondrial DNA (mtDNA) T16189C polymorphism, with a homopolymeric C-tract of 10–12 cytosines, is a putative genetic risk factor for idiopathic dilated cardiomyopathy in the African and British populations. We hypothesized that this variant may predispose to dilated cardiomyopathy in people who are infected with the human immunodeficiency virus (HIV).

**Methods:**

A case-control study of 30 HIV-positive cases with dilated cardiomyopathy and 37 HIV-positive controls without dilated cardiomyopathy was conducted. The study was confined to persons of black African ancestry to minimize confounding of results by population admixture. HIV-positive patients with an echocardiographically confirmed diagnosis of dilated cardiomyopathy and HIV-positive controls with echocardiographically normal hearts were studied. Patients with secondary causes of cardiomyopathy (such as hypertension, diabetes, pregnancy, alcoholism, valvular heart disease, and opportunistic infection) were excluded from the study. DNA samples were sequenced for the mtDNA T16189C polymorphism with a homopolymeric C-tract in the forward and reverse directions on an ABI3100 sequencer.

**Results:**

The cases and controls were well matched for age (median 35 years versus 34 years, P = 0.93), gender (males 60% vs 53%, P = 0.54), and stage of HIV disease (mean CD4 T cell count 260.7/μL vs. 176/μL, P = 0.21). The mtDNA T16189C variant with a homopolymeric C-tract was detected at a frequency of 26.7% (8/30) in the HIV-associated cardiomyopathy cases and 13.5% (5/37) in the HIV-positive controls. There was no significant difference between cases and controls (Odds Ratio 2.33, 95% Confidence Interval 0.67–8.06, p = 0.11).

**Conclusion:**

The mtDNA T16189C variant with a homopolymeric C-tract is not associated with dilated cardiomyopathy in black African people infected with HIV.

## Background

It is well-established that a proportion of patients who are infected with the human immunodeficiency virus (HIV) develop cardiomyopathy during the natural history of the retroviral disease [1]. A recent study from Rwanda has shown that the prevalence of HIV-associated cardiomyopathy is 17.7% in an African outpatient population, with low socio-economic status, longer duration of HIV infection, advanced HIV disease, and low plasma selenium levels being the main predisposing factors [2]. It is not known, however, whether genetic factors play any role in the predisposition to dilated cardiomyopathy in people who are infected with HIV [3].

A major advance in the study of the pathogenesis of idiopathic dilated cardiomyopathy has been the demonstration that 20–30% of cases are familial, suggesting that genetic factors may be involved in the aetiology of the disease [4]. In cases of sporadic dilated cardiomyopathy, genetic factors might confer increased susceptibility to disease in association with other environmental factors such as viral myocarditis. In addition to nuclear DNA abnormalities, mutations in the mitochondrial DNA (mtDNA) are known to contribute to the development of cardiomyopathy [5]. The myocardium is dependent on mitochondria for cellular oxidative phosphorylation and is, therefore, susceptible to genetic defects that affect mitochondrial function.

The importance of genetic risk factors is underscored by the reported association between the mtDNA T16189C variant with a homopolymeric C-tract in African and British populations [6]. Nucleotide T16189 lies in the middle of a homopolymeric-C tract where the transition from a thymine (T) to a cytosine (C) causes a homopolymeric C-tract of 10–12 bp in the displacement loop (D-loop) region of the mtDNA.

As expected, the association data of Khogali *et al *imply that the variant increases susceptibility to idiopathic dilated cardiomyopathy, but the presence of the variant is not sufficient to cause disease. The mtDNA T16189C variant maps precisely to a novel point of origin of mtDNA replication (OriB), which makes it likely that the variant will alter mitochondrial DNA function [7]. Therefore, the critical position of the T16189C variant in the mitochondrial genome suggests that the variant might have a direct role in the pathogenesis of dilated cardiomyopathy. Indeed, the T16189C variant with a 10–12 base pair homopolymeric C tract appears to be a genetic risk factor for other disorders, such as diabetes mellitus [8], and low birth weight [9]and is thus of medical interest.

A proportion of cases of idiopathic dilated cardiomyopathy is thought to be due to previous viral myocarditis [10]. We postulated that the mtDNA T16189C variant might predispose to dilated cardiomyopathy in people with a virus-associated form of cardiomyopathy, such as HIV-associated cardiomyopathy. We tested this hypothesis in a case-control study.

## Methods

### Study design

This was a case-control study of 30 cases of HIV-associated cardiomyopathy and 37 HIV-positive patients with echocardiographically normal hearts. Spurious associations may arise in genetic association studies due to the inclusion of a population sub-group having different allele frequencies for the variant under study [11]. To control for this source of confounding, the cases and controls were drawn from people of black African ancestry. The study was approved by the Research Ethics Committee of the University of Cape Town, and all participants gave written informed consent.

A commercial kit (Qiagen, DNA blood minikit, Southern Cross, USA) was used to isolate total DNA from blood samples according to standard methods. The DNA was analysed for the mtDNA T16189C variant with a homopolymeric C-tract using standard PCR and sequencing of the D-loop region (nucleotides 15894–16401) in both the forward and reverse directions [6]. The resultant amplicon was sequenced on the ABI3100 and analysed.

### Statistics

Logistic regression models were fitted to assess the association between HIV-associated cardiomyopathy and the mtDNA T16189C variant with a homopolymeric C-tract; a p-value of <0.05 was considered significant. Based on the realised number of cases and controls, the statistical power of the study was 80% (β = 0.20) based on a 90% level of significance (α = 0.10).

## Results

The clinical characteristics of the cases and controls are summarised in Table 1. We enrolled 30 cases of HIV-associated cardiomyopathy. Thirty seven HIV-positive controls with normal echocardiography examination were identified. The cases and controls were well matched for age, gender, and stage of HIV disease as determined by CD4 T cell count and HIV viral load (Table 1).

**Table 1:** Clinical characteristics of the HIV-associated cardiomyopathy cases and HIV-positive controls

	**HIVAC**	**HIV positive controls**	**P value**
Median Age (IQR, ± Stdev)	35 (27–41, ± 9.3)	34 (30–38, ± 7.7)	0.93
			
Gender			
Male	18 (60%)	20 (52.6%)	0.54*
female	12 (40%)	17 (45.9%)	
Mean LVEF (± Stdev)	33.4 (± 11.8)	66.7 (± 11.3)	0.0001
Mean LVIDd (± Stdev)	5.9 (± 0.88)	4.4 (± 0.50)	0.0001
Mean CD4 count (± Stdev)	260.7 (± 213.8)	176.1 (± 152.7)	0.21
RNA viral load (median)	680,774.2 (92,000)	231,574.8 (66,000)	0.16
Number	30	37	

HIVAC, HIV-associated cardiomyopathy; IQR, inter-quartile range; Stdev, standard deviation; * Mann-Whitney non-parametric test; LVIDd: left ventricular internal dimension in diastole

We found the mtDNA T16189C variant with a homopolymeric C-tract occurred at a frequency of 26.7% (8/30) in the HIV-associated cardiomyopathy cases and in 13.5% (5/37) of the HIV-positive controls with no cardiomyopathy (Table 2). When analyzing the T16189C transition alone, without regard to the presence or absence of an uninterrupted C-tract, we found the variant to occur at a frequency of 70% (21/30) in the HIV-associated cardiomyopathy cases and 62.2% (23/37) of the HIV-positive controls with no cardiomyopathy.

**Table 2:** Frequency of other polymorphisms in the 10 bp D-loop region (16184 bp–16193 bp) in cases and controls

	16184 bp – 16193 bp	**HIVAC (%)**	**HIV+ (%)**	**X^2^**	**P value**	**OR**
	
**Wild Type**	CCCCCTCCCCC					
**T16189C**	**- - - - - C - - - -**	21 (70)	23 (62.2)	0.44	0.50	1.42 (0.45–4.49)
						
Homopolymeric length variants		8 (30)	5 (13.5)	1.81	0.18	2.33 (0.67–8.06)
(a) length variant C -10	AAAA CCCCCCCCCC	7 (23)	3 (8)	2.98	0.08	3.45 (0.68–22.40)
(b) length variant C -11	AAAC CCCCCCCCCC	1 (3)	1 (2.7)	0.33	1.0	1.24 (0.02–100.11)
(c) length variant C -12	AACC CCCCCCCCCC	0	1 (2.7)	0.01	1.0	0.0 (0.0–48.10)
						
**Cohort Total**		30	37			

bp, base-pairs; %, Percentage; X^2^, chi square; OR, odds ratio

**Figure 1:**
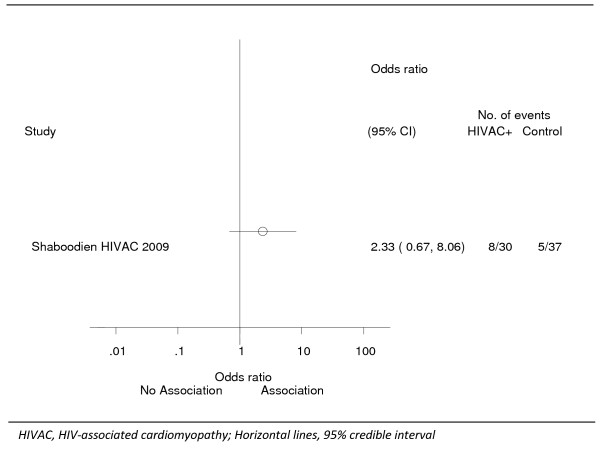
**Forest plot indicating the frequency of the mtDNA T16189C variant with a homopolymeric C-tract in HIV-associated cardiomyopathy patients and HIV-positive controls**.

There was no significant statistical association (Figure 1) between the mtDNA T16189C variant with a homopolymeric C-tract (Odds ratio 2.33, 95% Confidence interval 0.67 – 8.06) or the T16189C variant alone (Odds ratio 1.42, 95% Confidence interval 0.45 – 4.49) with HIV-associated cardiomyopathy (Table 2).

## Discussion

To the best of our knowledge, this is the first study of genetic risk factors in HIV-associated cardiomyopathy. This study found no significant association between the mtDNA T16189C variant with a homopolymeric C-tract and HIV-associated cardiomyopathy. However, there are differences between this study and the original report that found an association between the mtDNA T16189C variant with a homopolymeric C-tract and dilated cardiomyopathy [6]. Khogali *et al *studied patients with idiopathic dilated cardiomyopathy who were not tested for HIV, whereas we studied cases of HIV-associated cardiomyopathy and HIV infected controls. It is likely that some cases of idiopathic dilated cardiomyopathy result from unrecognized viral myocarditis early in life, a factor that led us to postulate that the same variant may predispose to an inflammatory cardiomyopathy such as HIV-associated cardiomyopathy. Idiopathic dilated cardiomyopathy is likely to represent a more heterogeneous group than patients with HIV-associated cardiomyopathy.

In the Khogali study, the finding of an association between the mtDNA T16189C variant containing a homopolymeric C-tract with idiopathic dilated cardiomyopathy in black Africans was based on 22 cases and 19 controls, a sample size that is smaller than the present study. It is well recognised that small gene association studies may be associated with false-positive findings [12]. The putative genetic association of the ACE I/D polymorphism with myocardial infarction is a salutary case in this regard. A series of smaller studies showed extreme, *albeit *conflicting findings of an association between ACE I/D genotype and myocardial infarction [13]. A larger, and adequately powered validation study was required to produce a more realistic estimate of the effect of the ACE I/D polymorphism on the risk of myocardial infarction; indeed a study involving thousands of cases and thousands of control showed no significant association between the ACE I/D polymorphism and the risk of myocardial infarction [13].

In the present study, it is uncertain whether the lack of significant difference between the cases and controls is related to a real absence of an association between the mtDNA T16189C variant with a homopolymeric C-tract and HIV-associated cardiomyopathy (i.e true-negative) or, due to inadequate statistical power of the present study (i.e false-negative). It is of interest that the original study of Khogali *et al *has not been replicated by other researchers, which suggests that the putative association of the mitochondrial T16189C with dilated cardiomyopathy requires independent validation. Other studies investigating this question have only addressed the frequency of the T16189C single nucleotide polymorphism and not the accompanying homopolymeric tract [14].

## Conclusion

We found that the mitochondrial T16189C variant with a homopolymeric-C tract was not associated with an increased risk of HIV-associated cardiomyopathy. Our finding therefore, raises concern about the validity of the putative association between the T16189C variant and cardiomyopathy in the original small study by Khogali [6]. One way of resolving this uncertainty is to conduct a validation study in an adequately powered study of cases and controls with dilated cardiomyopathy, and to pool the findings with all relevant studies in a meta-analysis [15].

## Competing interests

Financial competing interests

In the past five years we have not received reimbursements, fees, funding, or salary from any organization that may in any way gain or lose financially from the publication of this manuscript, either now or in the future.

We do not hold any stocks or shares in an organization that may in any way gain or lose financially from the publication of this manuscript, either now or in the future.

We do not hold nor are we currently applying for any patents relating to the content of the manuscript. We have not received reimbursements, fees, funding, or salary from an organization that holds or has applied for patents relating to the content of the manuscript.

We do not have any other financial competing interests.

Non-financial competing interests

We have no non-financial competing interests (political, personal, religious, academic, intellectual, commercial or any other) to declare in relation to this manuscript.

## Authors' contributions

BMM conceived the genetic study and designed the study with JP. GS performed the mutation analysis under the direction of BMM and JP. BMM and FFS conducted the phenotyping of cases and controls. MB and MEE conducted the statistical analysis. All authors contributed to the writing of the manuscript and have approved of the final draft that has been submitted for publication.

## Pre-publication history

The pre-publication history for this paper can be accessed here:


